# Performance of existing and novel surveillance case definitions for COVID-19 in household contacts of PCR-confirmed COVID-19

**DOI:** 10.1186/s12889-021-11683-y

**Published:** 2021-09-25

**Authors:** Hannah E. Reses, Mark Fajans, Scott H. Lee, Charles M. Heilig, Victoria T. Chu, Natalie J. Thornburg, Kim Christensen, Sanjib Bhattacharyya, Alicia Fry, Aron J. Hall, Jacqueline E. Tate, Hannah L. Kirking, Scott A. Nabity, The U.S. COVID-19 Household Investigation Team, Michelle Banks, Katherine A. Battey, Alison M. Binder, Sean Buono, Rebecca J. Chancey, Ann Christiansen, Erin E. Conners, Trivikram Dasu, Patrick Dawson, Elizabeth Dietrich, Lindsey M. Duca, Angela C. Dunn, Victoria L. Fields, Garrett Fox, Brandi D. Freeman, Radhika Gharpure, Christopher Gregory, Tair Kiphibane, Rebecca L. Laws, Sandra Lester, Nathaniel M. Lewis, Perrine Marcenac, Almea M. Matanock, Lisa Mills, Henry Njuguna, Michelle O’Hegarty, Daniel Owusu, Lindsey Page, Lucia Pawloski, Eric Pevzner, Mary Pomeroy, Ian W. Pray, Elizabeth M. Rabold, Jared R. Rispens, Phillip Salvatore, Amy Schumacher, Cuc H. Tran, Jeni Vuong, Ashutosh Wadhwa, Ryan P. Westergaard, Sarah Willardson, Dongni Ye, Sherry Yin, Anna Yousaf

**Affiliations:** 1 COVID-19 Response Team, Centers for Disease Control and Prevention, Atlanta, GA USA; 2 Epidemic Intelligence Service, Centers for Disease Control and Prevention, Atlanta, GA USA; 3 Utah Department of Health, Salt Lake City, UT USA; 4 City of Milwaukee Health Department, Milwaukee, WI USA

**Keywords:** COVID-19, SARS-CoV-2, Surveillance, Symptoms, Syndromic, Sensitivity, Specificity, Predictive values, Diagnostic accuracy, Children, Adults

## Abstract

**Background:**

Optimized symptom-based COVID-19 case definitions that guide public health surveillance and individual patient management in the community may assist pandemic control.

**Methods:**

We assessed diagnostic performance of existing cases definitions (e.g. influenza-like illness, COVID-like illness) using symptoms reported from 185 household contacts to a PCR-confirmed case of COVID-19 in Wisconsin and Utah, United States. We stratified analyses between adults and children. We also constructed novel case definitions for comparison.

**Results:**

Existing COVID-19 case definitions generally showed high sensitivity (86–96%) but low positive predictive value (PPV) (36–49%; F-1 score 52–63) in this community cohort. Top performing novel symptom combinations included taste or smell dysfunction and improved the balance of sensitivity and PPV (F-1 score 78–80). Performance indicators were generally lower for children (< 18 years of age).

**Conclusions:**

Existing COVID-19 case definitions appropriately screened in household contacts with COVID-19. Novel symptom combinations incorporating taste or smell dysfunction as a primary component improved accuracy. Case definitions tailored for children versus adults should be further explored.

**Supplementary Information:**

The online version contains supplementary material available at 10.1186/s12889-021-11683-y.

## Background

Coronavirus disease 2019 (COVID-19) is caused by severe acute respiratory syndrome coronavirus 2 (SARS-CoV-2). The virus was first identified in a cluster of patients with atypical pneumonia in Wuhan, China, in December 2019 [1]. Since its emergence, the virus has spread globally, causing widespread infection and death. Following evidence of person-to-person transmission and a broader clinical spectrum of infections, case definitions for COVID-19 have been revised [2]. In the initial weeks of the pandemic, COVID-19 was labeled a pneumonia of unknown etiology, and many who presented to medical care had classic pneumonia-like symptoms such as fever, cough, and dyspnea. An exceptional variety of symptoms has since been reported for COVID-19, ranging from none or mild indistinct symptoms to invasive neurological disease and fulminant respiratory failure [3–7]. As is common in the early response phases to novel emerging pathogens, there is ongoing need to reassess and refine surveillance case definitions for COVID-19 based on new information. Changes to case definitions affect interpretation of surveillance data, as was demonstrated by substantially different prevalence estimates when China broadened the COVID-19 case definition early in its epidemic response [2].

A few studies have demonstrated the predictive value of symptom profiles in healthcare workers [4, 8, 9] and other populations potentially not necessarily representative of the general public [5, 10]. These studies are subject to other limitations, too. Some applied predictive models that included serum biomarkers and imaging [11, 12]. Obtaining this information may limit real-world capture of people with mild-to-moderate SARS-CoV-2 infection and may delay public health intervention. Further, few studies to date have examined symptom combinations exclusively. Respiratory pathogens routinely behave differently in children and adults, and this appears to be true for COVID-19 as well [13]. For example, an assessment of ambulatory case surveillance definitions for influenza demonstrated lower sensitivity among children less than 5 years of age [14]. Similar analyses across age strata are lacking for COVID-19. Reliable, age-stratified syndromic surveillance definitions would likely aid public health officials to scale up community contact tracing and develop protocols to safely operate various congregate venues, such as schools and workplaces, should unlimited, timely diagnostic testing be unavailable.

Dedicated symptom-based surveillance systems have been developed to track COVID-19 cases. These include the U.S. Council of State and Territorial Epidemiologists (CSTE) original (CSTE combination 1; released April 5, 2020) and revised (CSTE combination 2; released August 7, 2020) clinical criteria for reporting SARS-CoV-2 infection, and the original CDC COVID-19–like illness (CLI) definition (Table 1). Similarly, the Centers for Disease Control and Prevention (CDC) maintains a list of symptoms for priority SARS-CoV-2 testing. These COVID-19 case definitions and the priority testing symptom list are intended to capture as many persons with COVID-19 as possible with confirmatory testing. Finally, longstanding respiratory virus surveillance networks established to monitor influenza-like illnesses (ILI) and acute respiratory infection (ARI), which is used for community-based syndromic surveillance of respiratory syncytial virus by the World Health Organization (WHO), may be plausibly adaptable platforms for monitoring COVID-19. The performance characteristics and utility of these syndromic surveillance platforms for COVID-19 have not been well defined [5].

**Table 1:** Existing COVID-19 case definitions, respiratory illness surveillance case definitions, and derived compound symptom combinations assessed for diagnostic performance in a community cohort of 185 individuals with household COVID-19 exposure in Utah and Wisconsin, United States, March–May 2020

Case definition category	Case definition	Criteria
Existing COVID-19 case definitions	U.S. Centers for Disease Control and Prevention (CDC) COVID-19-like illness (CLI)^a^	Fever, cough, or shortness of breath
	CDC symptom list^b^	Fever or chills, cough, shortness of breath or difficulty breathing, fatigue, muscle or body aches, headache, new loss of taste or smell, sore throat, congestion or runny nose, nausea or vomiting, diarrhea
	U.S. Council of State and Territorial Epidemiologists (CSTE) COVID-19 original clinical criteria (CSTE combination 1)^c^	At least one of the following: cough, shortness of breath, or discomfort breathing, OR at least two of the following: fever, chills, rigors, myalgia, headache, sore throat, new olfactory disorder and taste disorders
	CSTE COVID-19 revised clinical criteria (CSTE combination 2)^d^	At least one of the following: cough, shortness of breath, discomfort breathing, new olfactory disorder, new taste disorder, or at least two of the following: fever, chills, rigors, myalgia, headache, sore throat, nausea or vomiting, diarrhea, fatigue, congestion or runny nose
Existing respiratory illness surveillance case definitions	Influenza-like illness (ILI)^e^	Fever AND cough and/or sore throat
	World Health Organization (WHO) acute respiratory infection (ARI) definition for community-based respiratory syncytial virus (RSV) surveillance^f^	At least one of the following: shortness of breath or cough, sore throat, or coryza
Derived compound symptom combinations^g^	Derived compound combination 1	Taste and/or smell dysfunction, OR one of the following: shortness of breath, myalgia, or fever or chills
	Derived compound combination 2	Taste and/or smell dysfunction or discomfort breathing, OR at least two of the following: shortness of breath, wheezing, or fever or chills
	Derived compound combination 3	Taste and/or smell dysfunction, OR at least two of the following: shortness of breath, wheezing, discomfort breathing, or fever or chills
	Derived compound combination 4	Taste and/or smell dysfunction, OR shortness of breath and fever or chills

^a^U.S. Centers for Disease Control and Prevention (CDC) COVID-19-like illness (CLI) definition was used to guide early diagnostic testing strategies from 17 January 2020–08 March 2020 (https://emergency.cdc.gov/han/han00426.asp)

^b^U.S. Centers for Disease Control and Prevention (CDC) list of symptoms that may indicate COVID-19 infection (https://www.cdc.gov/coronavirus/2019-ncov/symptoms-testing/symptoms.html). This symptom list was last updated on 13 May 2020

^c^U.S. Council of State and Territorial Epidemiologists (CSTE) original clinical criteria for COVID-19 reporting (https://cdn.ymaws.com/www.cste.org/resource/resmgr/2020ps/interim-20-id-01_covid-19.pdf). This interim position statement (Interim-20-ID-01) was approved on 05 April 2020 and was replaced by Interim-20-ID-02 on 07 August 2020

^d^U.S. Council of State and Territorial Epidemiologists (CSTE) revised clinical criteria for COVID-19 reporting (https://cdn.ymaws.com/www.cste.org/resource/resmgr/ps/positionstatement2020/interim-20-id-02_COVID-19.pdf). This interim position statement (Interim-20-ID-02) was approved on 07 August 2020 and replaced Interim-20-ID-01

^e^Influenza-like illness (ILI) outpatient visit information collected through the U.S. Outpatient Influenza-like Illness Surveillance Network (ILINet) (https://www.cdc.gov/flu/weekly/overview.htm#anchor_1539281266932). This collaborative effort between CDC, state and local health departments, and healthcare providers has been tracking patients with ILI since the 1997–1998 influenza season

^f^World Health Organization (WHO) acute respiratory infection (ARI) definition for community-based respiratory syncytial virus (RSV) surveillance (https://www.who.int/influenza/rsv/rsv_case_definition/en/). Last updated 04 February 2020

^g^Compound symptom combinations were derived from all symptoms recorded at any time prior to enrollment (and after the index case’s symptom onset date) through the end of the 14-day observation period

We aimed to describe the diagnostic performance of two existing case definitions for COVID-19, the CDC COVID-19 symptom list, and two longstanding viral respiratory disease surveillance definitions among persons with confirmed SARS-CoV-2 exposure, stratified between adults and children. We also aimed to derive novel, practical symptom combinations in the same population. We interpreted the results primarily within the framework of two core public health surveillance functions: 1) symptom-based screening of individuals to guide SARS-CoV-2 diagnostic testing, contact tracing, and community-based isolation and quarantine, and 2) estimating disease frequency in persons with documented SARS-CoV-2 exposure. For symptom screening, we considered the merits of novel combinations when unlimited, timely diagnostic testing is unavailable.

## Methods

### Study design and data collection

CDC collaborated with state and local health departments in the Milwaukee, Wisconsin and Salt Lake City, Utah metropolitan areas in the United States to identify and enroll a convenience sample of people with laboratory-confirmed SARS-CoV-2 infection and their household contacts from March 22 to April 22, 2020. Ours are secondary analyses of this household transmission investigation whose questionnaire and methods were previously published in detail [15, 16]. This activity was reviewed by CDC and was conducted consistent with applicable federal law and CDC policy. See e.g., 45 Code of Federal Regulations (CFR) part 46, 21 CFR part 56; 42 United States Code (USC) §241(d); 5 USC §552a; 44 USC §3501 et seq.

We administered questionnaires to household contacts to assess the presence of 15 symptoms during the 14 days prior to or at enrollment (day 0). Additionally, participants completed a daily symptom diary during days 1–14 after enrollment. We collected serum and upper respiratory specimens (i.e., both nasopharyngeal [NP] and anterior nares swabs) on day 0 and day 14. We additionally collected NP swabs at any interim date if any household contact newly developed or had worsening of any one of 15 symptoms consistent with COVID-19: nasal congestion or runny nose, sore throat, cough, chest pain, shortness of breath, discomfort while breathing, wheezing, headache, new loss of taste or smell, fever/chills, fatigue, muscle aches, diarrhea (≥3 loose stools per day), abdominal pain, or nausea/vomiting. The Milwaukee Health Department and Utah Public Health Laboratories tested the swabs using the CDC real-time Reverse Transcriptase Polymerase Chain Reaction (RT-PCR) assay for SARS-CoV-2 [17], and CDC tested sera using a CDC-developed SARS-CoV-2 enzyme-linked immunosorbent assay (ELISA) [18].

### Definitions

We defined a household contact to be a COVID-19 case if they had at least one specimen test positive for SARS-CoV-2 by RT-PCR. We classified persons < 18 years of age as children, and persons ≥18 years of age as adults. We combined all symptoms recorded at any time prior to enrollment (and after the index case’s symptom onset date) through the end of the 14-day observation period. We assessed individual symptoms, existing symptom combinations, and newly constructed symptom combinations for their association with SARS-CoV-2 test result by RT-PCR (Table 1). We asked enrollees to state whether they experienced any loss of taste and, separately, smell during the specified time period. For enrollees who responded yes to this question, we then asked whether the loss was partial or complete. We defined loss and/or dysfunction of taste or smell to include any level of loss, whether partial or complete. For ARI, we interpreted coryza as runny nose or nasal congestion.

### Analytic methods

We excluded household contacts from the main analysis if not present at enrollment or not completing the study procedures. Our analysis of combinations predictive of COVID-19 included all 15 symptoms surveyed. We formally described the diagnostic performance of each individual symptom, existing COVID-19 case definitions, respiratory illness case definitions, and newly constructed symptom combinations (Table 1). The goal of assessing symptom combinations was to accurately divide the population into two groups: those who tested positive for SARS-CoV-2 and those who tested negative. For a given combination, we calculated the association of the combination with respect to laboratory-confirmed SARS-CoV-2 infection, yielding the number of contacts who were true positive (TP) (i.e., positive symptom profile and positive test), false negative (FN) (i.e., negative symptom profile and positive test), false positive (FP) (i.e., positive symptom profile and negative test), or true negative (TN) (i.e., negative symptom profile and negative test). From these values, we calculated the symptom combination’s sensitivity (i.e., TP / [TP + FN]), specificity (i.e., TN / [TN + FP]), positive predictive value (PPV) (i.e., TP / [TP + FP]), negative predictive value (NPV) (i.e., TN / [TN + FN]), F-1 score (the harmonic mean of sensitivity and PPV), and Youden’s index ([sensitivity + specificity] – 100). To determine how well each definition would estimate prevalence in a syndromic surveillance system, we also calculated the difference in the number of positive symptom screens (i.e., TP + FP) from the actual number of contacts who tested positive by RT-PCR. We assessed combinations across all ages and in children and adults separately, and we reported all measures on the percentage scale. To assess variability in each performance measure, we constructed bias-corrected and accelerated bootstrap confidence intervals [19] over 10,000 pseudosamples constructed by resampling households with replacement. We reported 95% confidence intervals with two exceptions. For measures estimated at 100% in the observed sample, we omitted confidence intervals, because the pseudosamples could not exhibit any variability. For the difference in specificity and sensitivity between adults and children, we reported 97.5% confidence intervals (a Bonferroni correction) to allow for a 95% joint confidence level regarding the differences in each pair.

We adapted innovative methods previously applied in the low-resource context to derive a parsimonious symptom combination to prioritize diagnostic testing for tuberculosis [20]. We chose this approach to be as comprehensive as practical for COVID-19 in that it systematically assessed nearly every conceivable combination of symptoms. First, we searched over 245,000 combinations of between one and 15 symptoms (i.e., simple combinations of the form “at least *m* symptoms present out of *n* symptoms considered”). We gave greater weight to combinations with high F-1 score or high Youden’s index. We then conducted an exhaustive search using pairs of these “m–of–n” combinations (i.e., compound combinations) to allow for more nuanced combinations. We limited this second search to single combinations of no more than five symptoms, such that the number of total symptoms evaluated for a compound combination was never more than ten. We allowed each pair of combinations to be joined by the logical operators [AND] and [OR], yielding approximately 73 million unique combination pairs. After the search, we selected four combination pairs to include in the primary analysis based on diagnostic performance and parsimony. We measured diagnostic performance by F-1 score (higher being better). We measured parsimony by the total number of symptoms evaluated (fewer being better) (Table 1).

We performed all calculations in R 4.0.0 (R Core Team), Python 3.7 (Python Software Foundation), or both. To allow for parallel processing, the exhaustive combinatorial search and bootstrap confidence intervals were implemented on a scientific workstation with 24 logical cores and 64 GB of RAM. De-identified data and analytic scripts in R and Python are publicly available through a GitHub repository: https://github.com/scotthlee/covid-casedefs.

## Results

### Study population

We enrolled 199 contacts of index patients with laboratory-confirmed SARS-CoV-2 infections within 62 households. We excluded one contact who was not living in the home on the day of enrollment, one who was hospitalized at enrollment, and two who did not consent to have specimens collected. Ten contacts had negative RT-PCR and positive serology test results; they were also excluded from the primary analyses. Therefore, our analyses included the remaining 185 household contacts. The median time interval from index patient’s symptom onset date to enrollment was 10 days (interquartile range [IQR]: 7–13). About half (95; 51%) were female. 108 (58%) were Caucasian/white, 32 (17%) Latinx/Hispanic, 23 (12%) African American/black, 14 (8%) Asian, four (2%) Native American, and four (2%) multiracial. The median age was 22 years ([IQR]: 14–47), with 122 (66%) adults and 63 (34%) children. Thirty-three enrollees (18%) were over age 50 years and 6 (3%) were over age 65 years. Despite the relatively young age distribution, nearly one-third (55; 30%) had at least one underlying health condition. Among children, nine (14%) were < 5 years, 19 (30%) were 5–9 years, and 35 (56%) were 10–17 years of age. SARS-CoV-2 infection was detected by RT-PCR in 49 (27%) household contacts. Separated by age group, 35/122 (29%) adults and 14/63 (22%) children had laboratory-confirmed SARS-CoV-2 infection by RT-PCR. Among the 49 RT-PCR-positive contacts, most (45; 92%) also had a positive serology result, three had a negative serology result, and one was not tested by serology.

### Performance characteristics for individual symptoms (all ages pooled)

**Fig. 1:**
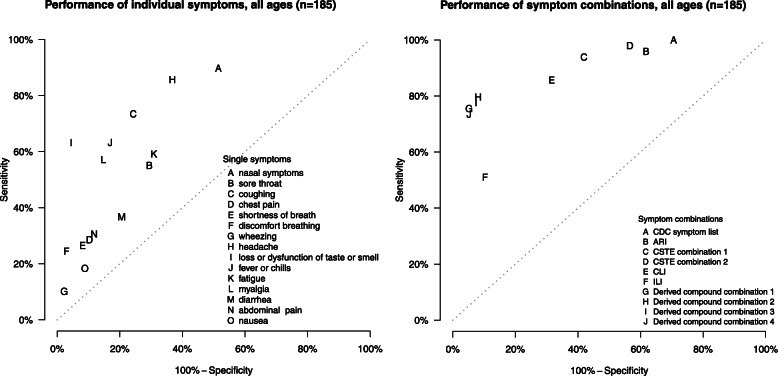
Sensitivity and 100%-specificity for individual COVID-19 symptoms, existing case definitions, and derived compound symptom combinations for a community cohort of 185 individuals with household exposure to COVID-19 in Utah and Wisconsin, United States, March–May 2020. Specificity is the probability of testing negative when disease is absent. Sensitivity is the probability of testing positive when disease is present. CDC symptom list=U.S. Centers for Disease Control and Prevention (CDC) list of symptoms that may indicate COVID-19 infection (https://www.cdc.gov/coronavirus/2019-ncov/symptoms-testing/symptoms.html). This symptom list was last updated on 13 May 2020. ARI=World Health Organization (WHO) acute respiratory infection (ARI) definition for community-based respiratory syncytial virus (RSV) surveillance (https://www.who.int/influenza/rsv/rsv_case_definition/en/). Last updated 04 February 2020. CSTE combination 1=U.S. Council of State and Territorial Epidemiologists (CSTE) original clinical criteria for COVID-19 reporting (https://cdn.ymaws.com/www.cste.org/resource/resmgr/2020ps/interim-20-id-01_covid-19.pdf). This interim position statement (Interim-20-ID-01) was approved on 05 April 2020 and was replaced by Interim-20-ID-02 on 07 August 2020. CSTE combination 2=U.S. Council of State and Territorial Epidemiologists (CSTE) revised clinical criteria for COVID-19 reporting (https://cdn.ymaws.com/www.cste.org/resource/resmgr/ps/positionstatement2020/interim-20-id-02_COVID-19.pdf). This interim position statement (Interim-20-ID-02) was approved on 07 August 2020 and replaced Interim-20-ID-01. CLI=U.S. Centers for Disease Control and Prevention (CDC) COVID-19-like illness (CLI) definition was used to guide early diagnostic testing strategies from 17 January 2020–08 March 2020 (https://emergency.cdc.gov/han/han00426.asp). ^††^ILI=Influenza-like illness (ILI) outpatient visit information collected through the U.S. Outpatient Influenza-like Illness Surveillance Network (ILINet) (https://www.cdc.gov/flu/weekly/overview.htm#anchor_1539281266932). This collaborative effort between CDC, state and local health departments, and healthcare providers has been tracking patients with ILI since the 1997–1998 influenza season. Derived compound combination 1: Taste and/or smell dysfunction, OR one of the following: shortness of breath, myalgia, or fever or chills. Derived compound combination 2: Taste and/or smell dysfunction or discomfort breathing, OR at least two of the following: shortness of breath, wheezing, or fever/chills. Derived compound combination 3: Taste and/or smell dysfunction, OR at least two of the following: shortness of breath, wheezing, discomfort breathing, or fever/chills. Derived compound combination 4: Taste and/or smell dysfunction, OR shortness of breath and fever/chills. Points closest to the upper left corner represent those with the highest sensitivity and specificity values

Individual symptoms with the highest sensitivity were nasal congestion or rhinorrhea, headache, and cough (Table 2, Fig. 1). Many of the individual symptoms reported were highly specific, although generally resulting in lower sensitivity. The exception was loss or dysfunction of taste or smell (categorized as a single symptom), which had a moderate sensitivity of 63% (95% confidence interval [CI] 47–77%), high specificity (96%; 95% CI 90–99%), high NPV (88%; 95% CI 80–93%), and the highest PPV (84%; 95% CI 64–94%), Youden’s index (59%; 95% CI 42–73%), and F-1 score (72%; 95% CI 57–83%).

**Table 2:** Diagnostic performance indicators for individual COVID-19 symptoms, existing case definitions, and derived compound symptom combinations for a community cohort of 185 people with household COVID-19 exposure in Utah and Wisconsin, United States, March–May 2020

	SARS-CoV-2-negative (*n* = 136)		SARS-CoV-2-positive (*n* = 49)		Specificity^a^		Sensitivity^b^		NPV^c^		PPV^d^		Youden’s index^e^		F-1 score^f^		Difference in prevalence^g^	
	TN^h^	FP^i^	FN^j^	TP^k^	Value	(95% CI)	Value	(95% CI)	Value	(95% CI)	Value	(95% CI)	Value	(95% CI)	Value	(95% CI)	Value	(95% CI)
**Individual symptoms**																		
*Upper respiratory*																		
Nasal congestion or rhinorrhea	66	70	5	44	49	(38, 59)	90	(80, 96)	93	(85, 97)	39	(27, 50)	38	(26, 50)	54	(42, 65)	133	(78, 227)
Sore throat	96	40	22	27	71	(61, 79)	55	(40, 68)	81	(72, 88)	40	(26, 56)	26	(10, 40)	47	(33, 59)	37	(0, 98)
*Lower respiratory*																		
Cough	103	33	13	36	76	(65, 84)	74	(60, 85)	89	(81, 94)	52	(37, 67)	49	(36, 62)	61	(49, 72)	41	(8, 94)
Chest pain	122	14	35	14	90	(83, 94)	29	(15, 42)	78	(69, 85)	50	(24, 70)	18	(5, 33)	36	(21, 51)	− 43	(−62, −15)
Shortness of breath	125	11	36	13	92	(86, 96)	27	(15, 40)	78	(67, 85)	54	(31, 75)	18	(6, 32)	36	(22, 49)	−51	(− 68, − 24)
Discomfort while breathing	132	4	37	12	97	(93, 99)	25	(14, 39)	78	(69, 85)	75	(40, 92)	22	(10, 36)	37	(22, 54)	−67	(−80, −49)
Wheezing	133	3	44	5	98	(94, 99)	10	(4, 21)	75	(65, 83)	63	(20, 100)	8	(1, 19)	18	(7, 33)	−84	(−92, −68)
*Neurological*																		
Headache	86	50	7	42	63	(52, 73)	86	(75, 93)	93	(85, 97)	46	(33, 59)	49	(35, 60)	60	(47, 70)	88	(44, 160)
Taste and/or smell dysfunction	130	6	18	31	96	(90, 99)	63	(47, 77)	88	(80, 93)	84	(64, 94)	59	(42, 73)	72	(57, 83)	−25	(−43, −5)
*Constitutional*																		
Fever or chills	113	23	18	31	83	(75, 89)	63	(49, 76)	86	(78, 92)	57	(40, 72)	46	(30, 60)	60	(46, 72)	10	(−15, 51)
Fatigue	94	42	20	29	69	(59, 79)	59	(41, 74)	83	(74, 89)	41	(25, 57)	28	(10, 44)	48	(33, 63)	45	(8, 106)
Myalgia	116	20	21	28	85	(77, 91)	57	(42, 70)	85	(77, 90)	58	(41, 74)	42	(28, 56)	58	(44, 69)	−2	(−28, 37)
*Gastrointestinal*																		
Diarrhea	108	28	31	18	79	(69, 88)	37	(20, 55)	78	(66, 86)	39	(22, 59)	16	(−3, 36)	38	(23, 54)	−6	(−40, 48)
Abdominal pain	120	16	34	15	88	(80, 94)	31	(19, 45)	78	(68, 85)	48	(27, 73)	19	(4, 35)	38	(23, 53)	−37	(−59, −2)
Nausea	124	12	40	9	91	(85, 95)	18	(10, 31)	76	(65, 83)	43	(20, 67)	10	(−1, 22)	26	(14, 40)	− 57	(−74, −30)
**Existing case definitions**																		
*COVID-19 and respiratory illness surveillance case definitions*																		
CDC symptom list^l^	40	96	0	49	29	(17, 45)	100	NA^w^	100	NA	34	(24, 45)	29	(17, 45)	51	(38, 62)	196	(122, 322)
ARI^m^	52	84	2	47	38	(26, 51)	96	(86, 100)	96	(88, 100)	36	(25, 48)	34	(23, 45)	52	(40, 64)	167	(102, 279)
CSTE combination 1^n^	79	57	3	46	58	(47, 69)	94	(82, 98)	96	(91, 99)	45	(32, 58)	52	(39, 63)	61	(47, 72)	110	(64, 190)
CSTE combination 2^o^	59	77	1	48	43	(35, 52)	98	(88, 100)	98	(90, 100)	38	(30, 47)	41	(32, 50)	55	(46, 64)	155	(110, 225)
CLI^p^	93	43	7	42	68	(57, 79)	86	(70, 94)	93	(88, 97)	49	(35, 64)	54	(40, 65)	63	(48, 75)	74	(35, 141)
ILI^q^	122	14	24	25	90	(83, 94)	51	(35, 65)	84	(75, 90)	64	(45, 79)	41	(25, 55)	57	(42, 69)	−20	(−41, 9)
**Derived compound symptom combinations**^r^																		
Combination 1^s^	129	7	12	37	95	(89, 98)	76	(60, 87)	92	(85, 96)	84	(67, 94)	70	(54, 82)	80	(67, 88)	−10	(−28, 9)
Combination 2^t^	125	11	10	39	92	(84, 97)	80	(65, 89)	93	(86, 96)	78	(59, 91)	72	(56, 83)	79	(66, 88)	2	(−17, 30)
Combination 3^u^	126	10	11	38	93	(85, 97)	78	(63, 88)	92	(86, 96)	79	(60, 91)	70	(54, 82)	78	(66, 87)	−2	(−20, 24)
Combination 4^v^	129	7	13	36	95	(89, 98)	74	(59, 84)	91	(84, 95)	84	(67, 94)	68	(54, 80)	78	(67, 86)	−12	(−29, 8)

^a^Specificity is the probability of testing negative when disease is absent

^b^Sensitivity is the probability of testing positive when disease is present

^c^*NPV* negative predictive value. NPV is the probability of a patient not having disease when test is negative

^d^*PPV* positive predictive value. PPV is the probability of a patient having disease when test is positive

^e^Youden’s index is defined as sensitivity plus specificity minus 100 (perfect score = 100)

^f^F-1 score is defined as the harmonic mean of sensitivity and PPV (perfect score = 100)

^g^Difference in prevalence is defined as the difference in the number of positive symptom screens (i.e., true positive plus false positive) for each combination from the actual number of contacts who tested positive by RT-PCR

^h^*TN* true negative

^i^*FP* false positive

^j^*FN* false negative

^k^*TP* true positive

^l^U.S. Centers for Disease Control and Prevention (CDC) list of symptoms that may indicate COVID-19 infection (https://www.cdc.gov/coronavirus/2019-ncov/symptoms-testing/symptoms.html). This symptom list was last updated on 13 May 2020

^m^World Health Organization (WHO) acute respiratory infection (ARI) definition for community-based respiratory syncytial virus (RSV) surveillance (https://www.who.int/influenza/rsv/rsv_case_definition/en/). Last updated 04 February 2020

^n^U.S. Council of State and Territorial Epidemiologists (CSTE) original clinical criteria for COVID-19 reporting (https://cdn.ymaws.com/www.cste.org/resource/resmgr/2020ps/interim-20-id-01_covid-19.pdf). This interim position statement (Interim-20-ID-01) was approved on 05 April 2020 and was replaced by Interim-20-ID-02 on 07 August 2020

^o^U.S. Council of State and Territorial Epidemiologists (CSTE) revised clinical criteria for COVID-19 reporting (https://cdn.ymaws.com/www.cste.org/resource/resmgr/ps/positionstatement2020/interim-20-id-02_COVID-19.pdf). This interim position statement (Interim-20-ID-02) was approved on 07 August 2020 and replaced Interim-20-ID-01

^p^U.S. Centers for Disease Control and Prevention (CDC) COVID-19-like illness (CLI) definition was used to guide early diagnostic testing strategies from 17 January 2020–08 March 2020 (https://emergency.cdc.gov/han/han00426.asp)

^q^Influenza-like illness (ILI) outpatient visit information collected through the U.S. Outpatient Influenza-like Illness Surveillance Network (ILINet) (https://www.cdc.gov/flu/weekly/overview.htm#anchor_1539281266932). This collaborative effort between CDC, state and local health departments, and healthcare providers has been tracking patients with ILI since the 1997–1998 influenza season

^r^Compound symptom combinations were derived from all symptoms recorded at any time prior to enrollment (and after the index case’s symptom onset date) through the end of the 14-day observation period

^s^Derived compound combination 1: Taste and/or smell dysfunction, OR one of the following: shortness of breath, myalgia, or fever or chills

^t^Derived compound combination 2: Taste and/or smell dysfunction or discomfort breathing, OR at least two of the following: shortness of breath, wheezing, or fever/chills

^u^Derived compound combination 3: Taste and/or smell dysfunction, OR at least two of the following: shortness of breath, wheezing, discomfort breathing, or fever/chills

^v^Derived compound combination 4: Taste and/or smell dysfunction, OR shortness of breath and fever/chills

^w^*NA* not applicable

### Performance characteristics for existing COVID-19 case definitions, the CDC symptom list, and respiratory syndromic Surveillance networks (all ages pooled)

Among the existing case definitions, sensitivity was perfect (100%) for the CDC symptom list definition, and also high for ARI (96%; 95% CI 86–100%), CSTE combination 1 (original); 94%; 95% CI 82–98%), CSTE combination 2 (revised); 98%; 95% CI 88–100%), and CLI (86%; 95% CI 70–94%) (Table 2, Fig. 1). While these definitions offered high sensitivity, they were poorly specific. Conversely, ILI was highly specific (90%; 95% CI 83–94%) but insensitive (51%; 95% CI 35–65%). All existing definitions demonstrated low PPV. Youden’s indices and F-1 scores were highest for CSTE combination 1 and CLI, though still suboptimal. None of the existing definitions predicted prevalence well; the difference from true prevalence ranged from − 20 for ILI to 196 for the CDC symptom list. Compared to CSTE combination 1, CSTE combination 2 had slightly higher sensitivity and NPV, but performed more poorly on all other diagnostic performance indicators. Although not included in the primary analyses, existing case definitions capture ≥90% of COVID-19 detected by ELISA only (Supplemental Table 1).

### Performance characteristics for derived compound symptom combinations (all ages pooled)

The four highest performing novel symptom combinations, based on F-1 score and parsimony, were compound symptom combinations that included dysfunction of taste or smell. These four combinations performed similarly well on all performance measures (Table 2, Fig. 1). We determined compound symptom combination 3 (i.e., loss or dysfunction of taste or smell, or at least two of the following: shortness of breath, wheezing, discomfort breathing, or fever/chills), to be simple to implement, have higher specificity (93%; 95% CI 85–97%), NPV (92%; 95% CI 86–96%), PPV (79%; 95% CI 60–91%), Youden’s index (70%; 95% CI 54–82%), and F-1 score (78%; 95% CI 66–87%) than existing case definitions. The compound symptom combination 3 showed near-perfect prevalence prediction (− 2; 95% CI − 20–24), and sensitivity was moderately high (78%; 95% CI 63–88%).

### Adult-child differences in discriminatory performance

**Fig. 2:**
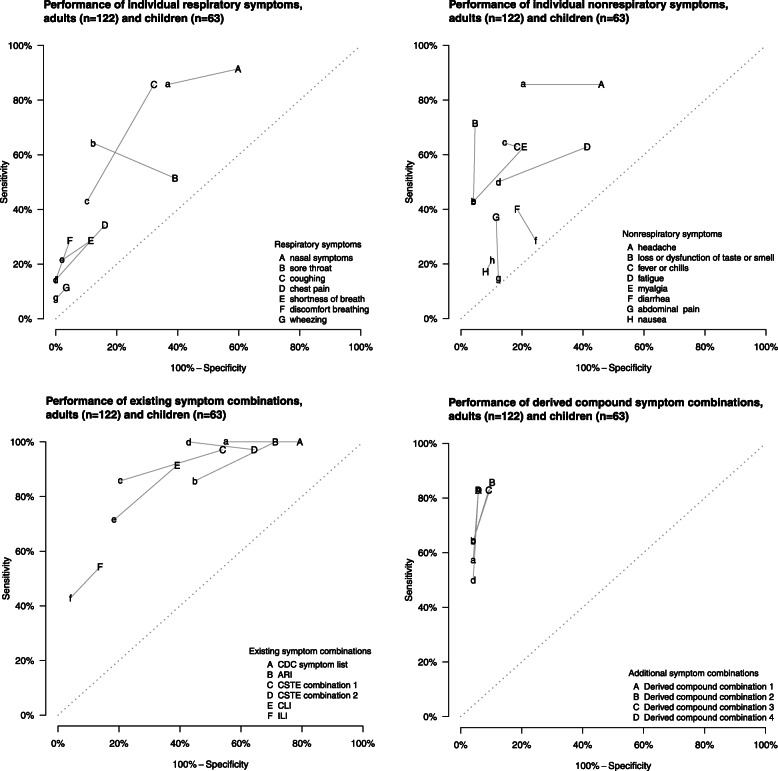
Sensitivity and 100%-specificity for individual COVID-19 symptoms, existing case definitions, and derived compound symptom combinations for a community cohort of 122 adults (upper case letters) and 63 children (lower case letters) with household exposure to COVID-19 in Utah and Wisconsin, United States, March–May 2020. Specificity is the probability of testing negative when disease is absent. Sensitivity is the probability of testing positive when disease is present. CDC symptom list=U.S. Centers for Disease Control and Prevention (CDC) list of symptoms that may indicate COVID-19 infection (https://www.cdc.gov/coronavirus/2019-ncov/symptoms-testing/symptoms.html). This symptom list was last updated on 13 May 2020. ARI=World Health Organization (WHO) acute respiratory infection (ARI) definition for community-based respiratory syncytial virus (RSV) surveillance (https://www.who.int/influenza/rsv/rsv_case_definition/en/). Last updated 04 February 2020. CSTE combination 1=U.S. Council of State and Territorial Epidemiologists (CSTE) original clinical criteria for COVID-19 reporting (https://cdn.ymaws.com/www.cste.org/resource/resmgr/2020ps/interim-20-id-01_covid-19.pdf). This interim position statement (Interim-20-ID-01) was approved on 05 April 2020 and was replaced by Interim-20-ID-02 on 07 August 2020. CSTE combination 2=U.S. Council of State and Territorial Epidemiologists (CSTE) revised clinical criteria for COVID-19 reporting (https://cdn.ymaws.com/www.cste.org/resource/resmgr/ps/positionstatement2020/interim-20-id-02_COVID-19.pdf). This interim position statement (Interim-20-ID-02) was approved on 07 August 2020 and replaced Interim-20-ID-01. CLI=U.S. Centers for Disease Control and Prevention (CDC) COVID-19-like illness (CLI) definition was used to guide early diagnostic testing strategies from 17 January 2020–08 March 2020 (https://emergency.cdc.gov/han/han00426.asp). ILI=Influenza-like illness (ILI) outpatient visit information collected through the U.S. Outpatient Influenza-like Illness Surveillance Network (ILINet) (https://www.cdc.gov/flu/weekly/overview.htm#anchor_1539281266932). This collaborative effort between CDC, state and local health departments, and healthcare providers has been tracking patients with ILI since the 1997–1998 influenza season. Derived compound combination 1: Taste and/or smell dysfunction, OR one of the following: shortness of breath, myalgia, or fever or chills. Derived compound combination 2: Taste and/or smell dysfunction or discomfort breathing, OR at least two of the following: shortness of breath, wheezing, or fever/chills. Derived compound combination 3: Taste and/or smell dysfunction, OR at least two of the following: shortness of breath, wheezing, discomfort breathing, or fever/chills. Derived compound combination 4: Taste and/or smell dysfunction, OR shortness of breath and fever/chills. Points closest to the upper left corner represent those with the highest sensitivity and specificity values

The accuracy of symptom profiles for defining RT-PCR confirmed COVID-19 differed by age (Table 3, Fig. 2). Overall, existing case definitions were less sensitive in children compared to adults. One exception, the CDC symptom list for priority testing (Table 1), captured all COVID-19 cases regardless of age. The existing case definitions were more specific in children, but the greater specificity was statistically significant for CSTE combination 1 only. Individual symptoms showed a similar pattern of lower sensitivity among children, notably taste/smell dysfunction. Sore throat was more sensitive in children, and fever/chills and nausea were similar regardless of age group. We observed a similar pattern of increased specificity for most derived symptom combinations in children (Table 3, Fig. 2). Cough was the sole symptom where the difference in both sensitivity and specificity was statistically significant. For both children and adults, the CLI case definition provided the greatest balance between both sensitivity and specificity (Youden’s Index 53%; 95% CI 8–80% vs. 52%; 95% CI 36–66%, respectively) and harmonization of sensitivity with PPV (F-1 61%; 95% CI 26–83% vs. 63%; 95% CI 49–76%, respectively) (Table 2). CLI also most accurately predicted overall prevalence amongst children (percent difference from true prevalence 36%; 95% CI –17–157) (Table 3).

**Table 3:** Diagnostic performance indicators for individual COVID-19 symptoms, existing case definitions, and derived compound symptom combinations by age group for 122 adults and 63 children with household exposure to COVID-19 in Utah and Wisconsin, United States, March–May 2020

	Adults								Children								Difference^e^			
	Specificity^a^		Sensitivity^b^		Youden’s index^c^		F-1 score^d^		Specificity		Sensitivity		Youden’s index		F-1 score		Specificity		Sensitivity	
	Value	(95% CI)	Value	(95% CI)	Value	(95% CI)	Value	(95% CI)	Value	(95% CI)	Value	(95% CI)	Value	(95% CI)	Value	(95% CI)	Value	(95% CI)^f^	Value	(95% CI)^f^
**Individual symptoms**																				
*Upper respiratory*																				
Nasal congestion or rhinorrhea	40	(30, 51)	91	(78, 97)	32	(17, 44)	54	(40, 66)	63	(44, 77)	86	(58, 100)	49	(25, 67)	55	(32, 71)	−23	(− 44, 2)	6	(−13, 31)
Sore throat	61	(50, 72)	51	(33, 68)	12	(−9, 31)	41	(26, 56)	88	(74, 96)	64	(29, 88)	52	(21, 78)	62	(35, 82)	−27	(−43, − 9)	−13	(− 47, 28)
*Lower respiratory*																				
Cough	68	(56, 78)	86	(71, 94)	54	(37, 68)	65	(51, 76)	90	(77, 96)	43	(13, 77)	33	(3, 68)	48	(18, 75)	−22	(−39, −5)	43	(2, 78)
Chest pain	84	(74, 91)	34	(18, 52)	18	(0, 38)	39	(22, 57)	100	NA^r^	14	(0, 29)	14	(0, 29)	25	(11, 40)	−16	(−27, −8)	20	(−6, 45)
Shortness of breath	89	(80, 94)	29	(16, 44)	17	(2, 34)	36	(21, 52)	98	(87, 100)	21	(0, 56)	19	(−2, 56)	33	(11, 63)	−9	(−20, 0)	7	(−33, 38)
Discomfort while breathing	95	(90, 99)	29	(13, 48)	24	(8, 43)	41	(21, 61)	100	NA	14	(0, 29)	14	(0, 29)	25	(11, 40)	−5	(−10, −1)	14	(−10, 40)
Wheezing	97	(91, 99)	11	(3, 25)	8	(−1, 21)	19	(6, 37)	100	(100, 100)	7	(0, 33)	7	(0, 33)	13	(6, 18)	−3	(−9, 0)	4	(−18, 18)
*Neurological*																				
Headache	54	(43, 65)	86	(71, 94)	40	(24, 53)	57	(44, 68)	80	(63, 90)	86	(63, 100)	65	(47, 81)	67	(44, 81)	−26	(−44, −4)	0	(−21, 24)
Taste and/or smell dysfunction	95	(89, 99)	71	(51, 85)	67	(47, 82)	78	(63, 88)	96	(84, 100)	43	(18, 67)	39	(13, 65)	55	(29, 76)	−1	(−8, 10)	29	(−3, 58)
*Constitutional*																				
Fever or chills	82	(72, 89)	63	(46, 78)	45	(27, 61)	60	(46, 73)	86	(68, 95)	64	(33, 90)	50	(14, 78)	60	(30, 82)	−4	(−20, 16)	−1	(−36, 37)
Fatigue	59	(47, 70)	63	(43, 78)	22	(1, 40)	47	(32, 61)	88	(71, 96)	50	(9, 80)	38	(1, 72)	52	(19, 79)	−29	(−48, −8)	13	(−32, 60)
Myalgia	79	(69, 87)	63	(45, 77)	42	(24, 58)	59	(44, 71)	96	(85, 100)	43	(11, 69)	39	(10, 64)	55	(23, 75)	−17	(−29, −4)	20	(−19, 62)
*Gastrointestinal*																				
Diarrhea	82	(71, 90)	40	(19, 60)	22	(0, 43)	43	(23, 62)	76	(55, 89)	29	(8, 60)	4	(−24, 37)	27	(10, 47)	6	(−13, 30)	11	(−27, 46)
Abdominal pain	89	(80, 94)	37	(21, 54)	26	(8, 44)	45	(27, 61)	88	(73, 97)	14	(0, 57)	2	(−17, 39)	18	(7, 45)	1	(−13, 17)	23	(−20, 49)
Nausea	92	(85, 96)	17	(6, 32)	9	(−3, 25)	25	(10, 43)	90	(77, 96)	21	(4, 62)	11	(−11, 50)	27	(9, 57)	2	(−8, 15)	−4	(−48, 23)
**Existing case definitions**																				
*COVID-19 and respiratory illness surveillance case definitions*																				
CDC symptom list^g^	21	(13, 32)	100	NA	21	(13, 32)	50	(38, 63)	45	(22, 65)	100	NA	45	(22, 65)	51	(29, 68)	−24	(−50, 6)	NA	NA
ARI^h^	29	(19, 41)	100	NA	29	(19, 41)	53	(40, 65)	55	(34, 72)	86	(58, 100)	41	(17, 60)	50	(29, 68)	−26	(−50, 0)	14	(0, 38)
CSTE combination 1^i^	46	(34, 58)	97	(81, 100)	43	(30, 55)	59	(45, 70)	80	(63, 91)	86	(43, 100)	65	(33, 83)	67	(38, 84)	−34	(− 53, −12)	11	(−7, 45)
CSTE combination 2^j^	36	(26, 46)	97	(84, 100)	33	(20, 44)	54	(44, 65)	57	(43, 70)	100	NA	57	(43, 70)	57	(39, 72)	−22	(−43, 0)	−3	(−14, 0)
CLI^k^	61	(48, 73)	91	(78, 98)	52	(36, 66)	63	(49, 76)	82	(64, 92)	71	(20, 95)	53	(8, 80)	61	(26, 83)	−21	(−41, 2)	20	(−10, 71)
ILI^l^	86	(76, 93)	54	(37, 70)	41	(23, 59)	58	(42, 71)	96	(85, 100)	43	(11, 80)	39	(7, 78)	55	(20, 85)	−10	(− 22, 2)	11	(−36, 53)
**Derived compound symptom combinations**^m^																				
Combination 1^n^	94	(88, 98)	83	(64, 94)	77	(58, 89)	84	(70, 92)	96	(84, 100)	57	(22, 83)	53	(17, 81)	67	(29, 86)	−2	(−10, 9)	26	(− 10, 64)
Combination 2^o^	90	(81, 96)	86	(67, 96)	75	(56, 87)	81	(67, 90)	96	(84, 100)	64	(38, 85)	60	(31, 83)	72	(43, 89)	−6	(−17, 5)	21	(−10, 51)
Combination 3^p^	91	(82, 96)	83	(64, 94)	74	(55, 86)	81	(66, 90)	96	(84, 100)	64	(38, 85)	60	(31, 83)	72	(43, 89)	−5	(−15, 5)	19	(−14, 49)
Combination 4^q^	94	(88, 98)	83	(64, 94)	77	(58, 89)	84	(70, 92)	96	(84, 100)	50	(17, 67)	46	(16, 69)	61	(30, 80)	−2	(−10, 9)	33	(3, 66)

^a^Specificity is the probability of testing negative when disease is absent

^b^Sensitivity is the probability of testing positive when disease is present

^c^Youden’s index is defined as sensitivity plus specificity minus 100 (perfect score = 100)

^d^F-1 score is defined as the harmonic mean of sensitivity and positive predictive value (perfect score = 100)

^e^Difference in specificity and sensitivity between adults and children

^f^Confidence intervals are jointly 95%, or 97.5% with a Bonferroni correction

^g^U.S. Centers for Disease Control and Prevention (CDC) list of symptoms that may indicate COVID-19 infection (https://www.cdc.gov/coronavirus/2019-ncov/symptoms-testing/symptoms.html). This symptom list was last updated on 13 May 2020

^h^World Health Organization (WHO) acute respiratory infection (ARI) definition for community-based respiratory syncytial virus (RSV) surveillance (https://www.who.int/influenza/rsv/rsv_case_definition/en/). Last updated 04 February 2020

^i^U.S. Council of State and Territorial Epidemiologists (CSTE) original clinical criteria for COVID-19 reporting (https://cdn.ymaws.com/www.cste.org/resource/resmgr/2020ps/interim-20-id-01_covid-19.pdf). This interim position statement (Interim-20-ID-01) was approved on 05 April 2020 and was replaced by Interim-20-ID-02 on 07 August 2020

^j^U.S. Council of State and Territorial Epidemiologists (CSTE) revised clinical criteria for COVID-19 reporting (https://cdn.ymaws.com/www.cste.org/resource/resmgr/ps/positionstatement2020/interim-20-id-02_COVID-19.pdf). This interim position statement (Interim-20-ID-02) was approved on 07 August 2020 and replaced Interim-20-ID-01

^k^U.S. Centers for Disease Control and Prevention (CDC) COVID-19-like illness (CLI) definition was used to guide early diagnostic testing strategies from 17 January 2020–08 March 2020 (https://emergency.cdc.gov/han/han00426.asp)

^l^Influenza-like illness (ILI) outpatient visit information collected through the U.S. Outpatient Influenza-like Illness Surveillance Network (ILINet) (https://www.cdc.gov/flu/weekly/overview.htm#anchor_1539281266932). This collaborative effort between CDC, state and local health departments, and healthcare providers has been tracking patients with ILI since the 1997–1998 influenza season

^m^Compound symptom combinations were derived from all symptoms recorded at any time prior to enrollment (and after the index case’s symptom onset date) through the end of the two-week observation period

^n^Combination 1: Taste and/or smell dysfunction, OR one of the following: shortness of breath, myalgia, or fever or chills

^o^Combination 2: Taste and/or smell dysfunction or discomfort breathing, OR at least two of the following: shortness of breath, wheezing, or fever/chills

^p^Combination 3: Taste and/or smell dysfunction, OR at least two of the following: shortness of breath, wheezing, discomfort breathing, or fever/chills

^q^Combination 4: Taste and/or smell dysfunction, OR shortness of breath and fever/chills

^r^*NA* = not applicable

## Discussion

Existing case surveillance definitions for COVID-19, as shown in Table 1, were generally sensitive in our study conducted among household contacts of infected persons, a population with proven SARS-CoV-2 exposure. However, they tended to have low specificity and poorly estimated disease prevalence. By systematically screening novel definitions that optimized sensitivity, specificity, and PPV, we improved community prevalence estimation and overall accuracy of individual screening, which could be useful if diagnostic testing is limited. In particular, we affirmed loss or dysfunction of taste or smell as a uniquely discerning characteristic central to constructing an effective, concise case surveillance definition when applied across all age groups (i.e., derived compound combination 3).

An appropriate discriminatory balance between sensitivity and specificity for a newly emerging pathogen depends on the objectives of the surveillance activity [21]. Highly sensitive case definitions capture a larger proportion of true COVID-19 cases, which is ideal when diagnostic testing is widely available and results are timely. Highly sensitive definitions, however, generally rule in a larger number of non-cases (i.e., FP symptom screens) [22]. In addition to testing resources, the public health system’s tolerance for false-positive screens is, of course, dependent on human resources. This is especially apparent when intensive interventions involve extensive contact tracing, isolation and quarantine. At high community COVID-19 prevalence, these intensive mitigation efforts may benefit from evidence-based prioritization. By example, CSTE combination 2 had a FP symptom screening rate (77/136; 57%) eight times that for derived compound combination 3 (10/136; 7%) in our cohort. At the population level, such differences could expose shortcomings in resources for core interventions, such as universal contact tracing. The COVID-19 response has repeatedly been strained in these requisite areas [23–25]. Novel symptom screening criteria that more tightly couple sensitivity and specificity (i.e., diagnostic accuracy), such as the derived compound combination 3, could help to prioritize interventions when strategically deployed. This principle may also apply when evaluating novel vaccines or therapeutics in large clinical trials involving thousands of participants, where feasibility constraints often dictate the use of symptom-prioritized testing to confirm outcomes. Still, highly sensitive symptom rules, such as CSTE combination 2, are preferred for COVID-19 when resources are unlimited.

For using syndromic surveillance systems to estimate community burden, the highly sensitive existing case definitions overestimated true burden. Conversely, highly specific case definitions, such as ILI, may detect changes in disease trends over time but underestimate true burden [21]. ILI underestimated disease prevalence by more than 80% in this study population. Current laboratory-based surveillance grossly under-ascertains incidence [26], especially where diagnostic testing is not easily accessible or widespread. Retailoring community-based syndromic surveillance systems already in place [27] (i.e., altering the symptoms included or applying a correction factor based on results such as ours) would more accurately reflect true burden.

For most symptoms and their combinations, overall performance, most notably sensitivity, differed between child and adult household contacts. These findings are consistent with prior observations whereby children generally show fewer and milder symptoms of COVID-19 compared with adults [28], and COVID-19 syndromes vary across ages [13]. The small number of children with COVID-19 in this cohort limits the conclusion of specific recommendations, but further examination into the utility of age-specific case definitions is warranted in considering policies for schools and other child congregate settings, and for deriving accurate burden estimates from syndromic surveillance.

While the number of individuals in this study is relatively small, our study population is well-characterized. We collected extensive symptom data, which yielded a comprehensive assessment of multiple symptom combinations. We also employed inclusion criteria that were not based on disease status or symptom status, and a reference category based on standardized laboratory testing. Nonetheless, we acknowledge this study’s limitations. These analyses were not intended to produce definitive symptom combinations to be applied to the general public, however they may be used to guide the development of future candidate case definitions. One key consideration for future validation efforts is that enrollment started immediately after the precipitous decline in laboratory-confirmed influenza virus infections in the United States in mid-March 2020 [29]. Thus, our estimates of diagnostic performance may differ during the viral respiratory season. In addition, COVID-19 prevalence was higher for our study population (i.e., contacts of laboratory-confirmed household members) compared to the entire community, thereby limiting the generalizability of predictive values (although sensitivity and specificity remain unaffected by disease prevalence). Our study population was younger than the general population and the screening criteria may perform differently in older adults. We did not have enough older household contacts to permit further stratification among adults. Finally, screening criteria applied to persons seeking medical care may also perform differently, as those individuals probably tend to have more severe illness.

Additionally, we showed that existing COVID-19 case definitions are highly sensitive and do well to screen in persons for testing and individual-level public health interventions like community isolation. In the first such endeavor for evaluating and deriving novel COVID-19 case surveillance definitions in a community setting among SARS-CoV-2–exposed individuals with largely mild illness, we evaluated novel symptom combinations for COVID-19 using methodology previously applied to tuberculosis in low resource settings [20]. These derived combinations and CSTE definition 2 better estimated disease burden and used taste and/or smell dysfunction as a primary component. The latter is supported by prior studies [5, 8–10]. Because most SARS-CoV-2 infections are mild [30] and core public health functions may need prioritization when testing and other resources are limited, case definitions that accurately determine COVID-19 status in the general public may assist continued interruption of community transmission [31]. When timely diagnostic testing is readily available, however, using less sensitive screening tools could inappropriately miss cases and lead to further community transmission.

Our study population, which includes participants enrolled independent of disease and symptom status, may better reflect the diagnostic performance in the general population than previously published research. Accurate clinical case definitions are likely to also apply to large clinical trials for candidate vaccines and therapeutics where serial confirmatory SARS-CoV-2 testing for any new symptom is impractical. It is important that our results be validated against the growing body of larger ambulatory surveillance databases in diverse communities and in other countries; in particular, our methodology should be assessed in the context of the annual influenza season, at varying community COVID-19 prevalence, and across the age spectrum. Such studies ideally can be accompanied by cost-effectiveness modeling of intervention strategies.

## Conclusions

The discriminatory performance of case surveillance definitions for COVID-19 is important for implementing effective epidemic mitigation strategies. Our study illustrates the performance of case definitions in community members with household exposure to SARS-CoV-2 based solely on symptom profiles. Prior work overrepresented healthcare workers or otherwise studied non-representative populations, and they did not examine both adults and children. Our study also provides a novel framework for refining definitions. Using 15 symptoms associated with COVID-19 for all contacts regardless of disease status, we systematically evaluated the discriminatory performance of individual symptoms and previously defined case surveillance definitions in adults and children, and according to two core surveillance applications: 1) screening non-hospitalized individuals to prioritize public health interventions, and 2) estimating the number of non-hospitalized persons with COVID-19 (i.e., community-based syndromic surveillance). We also constructed novel symptom combinations that effectively performed both functions and, in this study population, improved upon widely used case surveillance definitions that may help to target interventions in the absence of unlimited laboratory diagnostic capacity. Based on our results, case surveillance definition performance may increase if developed separately for adults and children.

## Supplementary Information

**Additional file 1: Supplemental Table 1.** Individual COVID-19 symptoms and existing case definitions by 2019-nCoV Real-Time RT-PCR assay and a SARS-CoV-2 spike protein enzyme-linked immunosorbent assay (ELISA) results in Utah and Wisconsin, United States, March–May 2020.

## Data Availability

De-identified data and analytic scripts in R and Python are publicly available through a GitHub repository: https://github.com/scotthlee/covid-casedefs.

## Abbreviations

COVID-19: Coronavirus disease 2019
PPV: Positive predictive value
SARS-CoV-2: Severe acute respiratory syndrome coronavirus 2
CSTE: Council of State and Territorial Epidemiologists
CLI: Coronavirus-like illness
CDC: United States Centers for Disease Control and Prevention
ILI: Influenza-like illness
ARI: Acute respiratory illness
WHO: World Health Organization
CFR: Code of Federal Regulations
USC: United States Code
NP: Nasopharyngeal
RT-PCR: Reverse transcriptase polymerase chain reaction
ELISA: Enzyme-linked immunosorbent assay
TP: True positive
FN: False negative
TN: True negative
FP: False positive
NPV: Negative predictive value
GB: Gigabytes
RAM: Random access memory
IQR: Interquartile range
CI: Confidence interval
